# Enhancing Electrospinnability of Chitosan Membranes in Low-Humidity Environments by Sodium Chloride Addition

**DOI:** 10.3390/md22100443

**Published:** 2024-09-27

**Authors:** Hengjie Su, Xiaoqi Chen, Linna Mao, Ting Li

**Affiliations:** 1 Institute of Biomedical Engineering, Chinese Academy of Medical Sciences & Peking Union Medical College, Tianjin 300192, China; 2 Department of Biomedical Engineering, Tiangong, Tianjin 300387, China

**Keywords:** chitosan, salt, electrospinning, environmental humidity, conductivity

## Abstract

The electrospinning of pure chitosan nanofibers is highly sensitive to environmental humidity, which limits their production consistency and applicability. This study investigates the addition of sodium chloride (NaCl) to chitosan solutions to enhance spinnability and mitigate the effigurefects of low humidity. NaCl was incorporated into the electrospun chitosan solution, leading to increased conductivity and decreased viscosity. These modifications improved the electrospinning process. Comparative analyses between chitosan membranes (CM) and sodium-chloride-added chitosan membranes (SCM) revealed no significant differences in chemical structure, mechanical strength, or in vitro cell proliferation. This indicates that the addition of 1% (*w*/*v*) NaCl does not adversely affect the fundamental properties of the chitosan membranes. The findings demonstrate that NaCl addition is a viable strategy for producing electrospun chitosan nanofibers in low-humidity environments, maintaining their physicochemical properties while enhancing spinnability.

## 1. Introduction

Electrospun chitosan membranes are being explored as biocompatible and biodegradable scaffolds for guided bone regeneration (GBR), with potential applications in periodontal treatment and craniofacial regeneration [1,2,3]. Chitosan, a nature polysaccharide derived from the shell of crustacean, offers advantages such as low cost, biocompatibility, biodegradability, and non-toxicity [4,5]. Consequently, it has been widely used in biomedical fields, including guided bone regeneration, vascular stents, and wound dressings [6,7]. Electrospinning is a method that uses an electrostatic field to create a jet from a polymer solution to form nanofibers [8]. Key factors for the successful spinning of nanofibers include viscosity, surface tension, and electrical conductivity of the solution [5]. These factors vary with changes in the solution concentration and solvent system.

Electrospinning pure chitosan nanofibers is challenging due to several inherent properties of chitosan. The solubility of chitosan in most common solvents is not ideal [9], and the solution viscosity, caused by strong intermolecular and intramolecular hydrogen bonds, makes it difficult to overcome the surface tension during electrospinning [10]. Acetic acid and trifluoroacetic acid (TFA) are widely used solvents for dissolving chitosan. Achieving suitable surface tension for electrospinning typically requires at least 30% acetic acid for chitosan with 56–65% deacetylation (DDA) [11]. TFA has been explored as a solvent because it can block the positive charges of amino groups on chitosan and decrease the electrostatic forces, enabling the production of homogeneous chitosan nanofibers [5]. When comparing the two solvents, TFA produces smoother and more continuous nanofibers than acetic acid [5]. Additionally, the addition of methylene chloride to TFA can result in more uniform fibers produced by electrospinning [12]. Although there have been concerns about the toxicity of TFA [13], studies have proved that electrospun chitosan membranes produced using TFA/DCM solvents are biocompatible both in vitro and in vivo [14,15]. Researchers continue to use TFA/DCM as the solvent for electrospinning chitosan due to its ability to produce a more controlled and stable electrospinning processes [16,17,18,19,20].

However, the success of electrospinning pure chitosan in TFA/DCM depends significantly on environmental moisture [13,21]. It has been observed that the ambient humidity suitable for chitosan electrospinning is between 40–60% [14,21]. The impact of environmental humidity on electrospinning is not fully understood, making pure chitosan electrospinning highly restricted to environmental humidity. It is suspected that lower humidity in the environment results in decreased electrostatic discharge on the spinning jet, as fewer water molecules are available for charge transfer [22]. Consequently, the charge density on the jet is higher at low humidity, causing fiber breakage. If the voltage applied to the electrostatic field is not high enough, or if the solution has low conductivity, the electrostatic force cannot overcome the surface tension, and fibers cannot be produced [23]. To address this, creating a humid atmosphere within the electrospinning apparatus has been explored. However, the dense water mist from the humidifier may cause short circuits in the high voltage electrostatic field, making it difficult to control the mist distribution and posing significant safety risks.

Another approach to improve spinnability is by enhancing the electrical conductivity of the electrospinning solution. In one study, adding tetraethylammonium bromide (TEAB) salt to a polyimides (PI) solution improved its conductivity, allowing successful spinning even at low PI concentrations [8]. Similarly, adding TEAB salt to a polymers of intrinsic microporosity (PIM-1) solution significantly increased its conductivity, enabling the formation of electrospun fibers at a low PIM-1 concentration (10% (*w*/*v*)) [24]. Salt ions act as charge carriers in the electric field and affect the motion of the spinning jet [25,26,27]. Although adding salt to electrospinning solutions to improve spinnability has been explored, no studies have focused on adding salt specifically to pure chitosan solutions for electrospinning. Additionally, the relationship between environmental humidity and the addition of salt to chitosan solutions has not been explored.

This study aims to address the issue of humidity constraints encountered during the electrospinning of chitosan using the TFA/DCM solvent combination. In this study, adding sodium chloride to the electrospun chitosan solution was explored to reduce the impact of environmental moisture. To further investigate the effect of salt on electrospun chitosan membranes, the physical and chemical properties of the electrospun chitosan solution and chitosan membrane (CM) were examined by comparing them with the electrospun chitosan solution with salt and chitosan membrane with salt (SCM). 

## 2. Results and Discussion

### 2.1. Solution Conductivity and Viscosity

**Figure 1:**
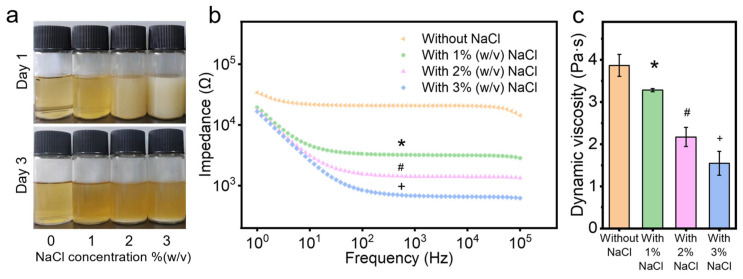
Properties of chitosan solutions with and without NaCl: (**a**) images of electrospun chitosan solutions on day 1 and day 3, (**b**) impedance, and (**c**) dynamic viscosity. *, #, and + denotes significant differences between groups.

To prepare the chitosan solution with NaCl, 5.5% (*w*/*v*) chitosan was dissolved in 10 mL TFA/DCM (7:3 *v*/*v*) solution overnight [14,28]. Just before electrospinning, 100 mg of NaCl was dissolved in 300 μL of water and added to the chitosan solution. Given that the water solubility of NaCl is 360 g/L and NaCl is hardly soluble in TFA and DCM, the NaCl solution was added to the chitosan solution in this specified proportion. NaCl was selected to increase the solution’s conductivity due to its biocompatibility as a natural component of the human body. Due to the immiscibility of water and DCM, the system separates into two phases after adding the NaCl solution to the electrospun chitosan solution: the TFA/water phase and the TFA/DCM phase. However, the TFA/water phase is very small and nearly invisible to the naked eye due to the tiny volume of water relative to the total volume. The NaCl dissolves in the TFA/water phase but not in the TFA/DCM phase. Given the small amount of water (300 μL) in a much larger volume of TFA/DCM (10 mL), the dissolution of NaCl is limited. This was confirmed in Figure 1a. After three days of deposition of the electrospun chitosan solutions with varying concentrations of NaCl, salt crystals were observed at the bottom of the solutions, indicating that the NaCl was relatively isolated from the electrospun solution (Figure 1a). Additionally, observations on the first day revealed that the mixed state of NaCl in the chitosan solution remained stable during the electrospinning process.

An imperative step in electrospinning involves balancing the charge behavior and viscosity of the solution [28]. The charge behavior impacts the spinnability of the solution, as higher solution conductivity enables greater splaying ability to eject the solution into filaments from the Taylor cone [29,30]. Adding an NaCl water solution can enhance the conductivity of the solution, improving the electrospinning process by reducing the impedance and potentially increasing spinnability [31,32]. This is consistent with the results of this study, where the impedance of the chitosan solution significantly decreased (*p* < 0.05) after adding salt (Figure 1b). Chitosan solution with higher salt concentrations showed lower impedance, indicating higher conductivity and potentially greater spinnability. 

The viscosity measures the resistance or internal friction of a fluid to flow, greatly influencing the electrospinning process and resulting fiber diameters [28,33]. After adding the NaCl solution, the dynamic viscosity of electrospun chitosan solutions decreased significantly (*p* < 0.05, Figure 1c). These results are consistent with Varnaitė-Žuravliova’s research, which showed that conductivity increased and viscosity decreased after adding NaCl to the chitosan/PEO electrospun solution [25]. Previous studies demonstrated that adding NaCl to chitosan solution can lead to the breakdown of the hydrogen bond network of chitosan [25,31,34,35]. The hydroxyl group of chitosan is usually involved in the formation of intermolecular hydrogen bonds, and the destruction of these hydrogen bonds promotes the molecular motion of chitosan, increases the charge density, and decreases the solution viscosity, and thus, enhances spinnability [31,34]. The optimal viscosity range can vary depending on the specific polymer and solvent system used [11,36,37,38,39,40]. When the viscosity is lower than the optimal range, it is difficult to draw continuous nanofibers, resulting in the appearance of beads in the membrane [11]. The viscosity of 10 mL electrospun chitosan solution with 300 mg (3% (*w*/*v*)) NaCl decreased below 2 Pa·s (Figure 1c), making it challenging to perform the electrospinning at this viscosity. In lab experiments, it was demonstrated that adding 100 mg (1% (*w*/*v*)) and 200 mg (2% (*w*/*v*)) NaCl to 10 mL of electrospun chitosan solution allowed successful electrospinning under the environmental moisture levels of 25–35%. However, it was difficult to collect chitosan membranes with 2% (*w*/*v*) NaCl at the same volume solution, possibly because the increased amount of water melted the fibers during the process, thereby decreasing the final fiber volume. Hence, the most suitable concentration of NaCl to add in the chitosan solution is 1% (*w*/*v*).

### 2.2. Scanning Electron Microscopy (SEM)

**Figure 2:**
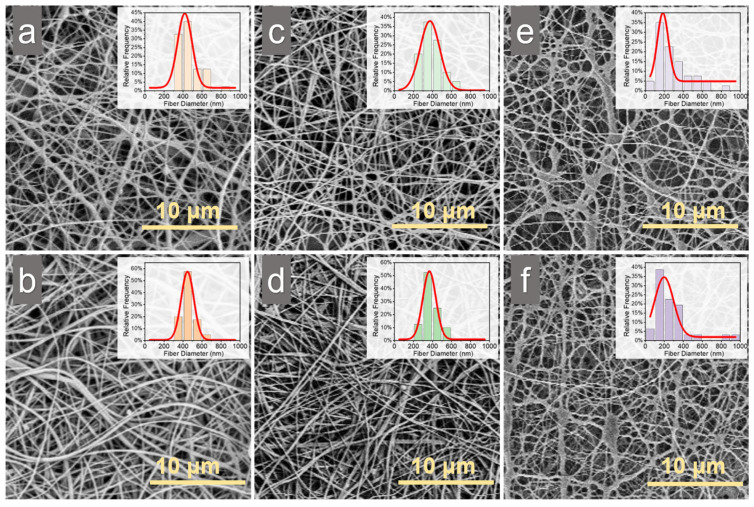
SEM images of electrospun chitosan membranes and the corresponding fiber diameter distribution: (**a**) CM without post-treatment, (**b**) CM with TEA/tBOC treatment, (**c**) SCM (1% NaCl) without post-treatment, (**d**) SCM (1% NaCl) with TEA/tBOC treatment, (**e**) SCM (2% NaCl) without post-treatment, and (**f**) SCM (2% NaCl) with TEA/tBOC treatment. “CM” denotes “chitosan membrane”, while “SCM” denotes “chitosan membrane with salt”.

**Figure 3:**
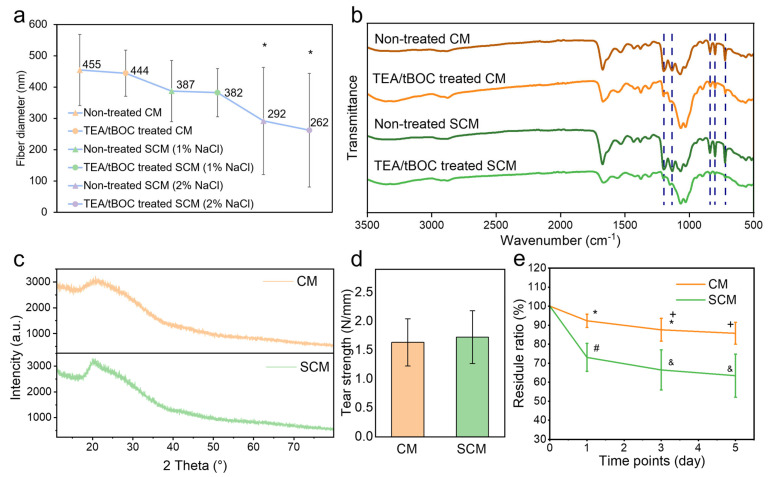
Physicochemical properties of electrospun chitosan membranes: (**a**) fiber diameter, (**b**) FTIR spectra, (**c**) XRD spectra, (**d**) tear strength, and (**e**) degradation ratio results. *, +, #, and & denote significant differences between groups.

After collecting the electrospun membranes, a post electrospinning treatment involving triethylamine (TEA) and di-tert-butyl dicarbonate (tBOC) was performed to neutralize the membranes, enhancing their aqueous stability and biocompatibility. SEM images showed that both SCM and CM maintained intact fiber structures before and after TEA/tBOC treatment, suggesting that the addition of the NaCl solution did not affect the uniformity of the spinning process, even with the formation of liquid phases (Figure 2). The diameter of SCM (with 2% (*w*/*v*) NaCl) fiber was significantly smaller (*p* < 0.05) than that of CM and SCM (with 1% (*w*/*v*) NaCl) before and after TEA/tBOC treatment (Figure 3a). The fiber diameter distribution showed that most fiber diameters of CM and SCM with 1% (*w*/*v*) NaCl were around 400 nm, whereas the majority of fiber diameters of SCM with 2% (*w*/*v*) NaCl were around 200 nm (Figure 2). Although there was no significant difference in fiber diameters between CM and SCM (with 1% (*w*/*v*) NaCl), a decreasing trend from CM to SCM (with 1% (*w*/*v*) NaCl) was observed. These results indicate that the addition of NaCl to the chitosan electrospinning solution reduces fiber diameter, but small amounts of salt do not significantly affect the fiber diameter. This aligns with findings that fiber diameter is influenced by the viscosity of the electrospun solution [40,41,42]. In this study, the addition of NaCl significantly decreased the viscosity of the electrospun chitosan solution, resulting in smaller diameters of electrospun chitosan nanofibers. Concurrently, the conductivity of the electrospun chitosan solution increased with the addition of NaCl, indicating an increase in charge density on the jet. This increased the extension level of the jet under the electric field [26]. Therefore, under the same applied voltage and spinning distance, higher solution conductivity enhances the jet’s axial stretch, resulting in smaller electrospun fiber diameters [43], see more in Supplementary Materials. 

After post-electrospinning treatment, there was no significant change (*p* > 0.05) in fiber diameter of both CM and SCM membranes. This is consistent with previous studies showing that post-electrospinning treatment with TEA/tBOC does not cause fiber expansion or structural loss [14].

### 2.3. Fourier Transform Infrared Spectroscopy (FTIR)

The FTIR spectra of CM and SCM (with 1% (*w*/*v*) NaCl) before and after TEA/tBOC treatment are compared in Figure 3b. The peaks associated with TFA salt at 723 cm^−1^, 801 cm^−1^, and 840 cm^−1^ disappeared after the TEA/tBOC treatment of both CM and SCM membranes, which is consistent with the previous study [14]. Additionally, two peaks around 1100–1200 cm^−1^ disappeared after the TEA/tBOC treatment, indicating the removal of trifluoromethyl groups. 

The presence of NaCl in the FTIR spectrum was not clearly evident. There were hardly any characteristic bands related to NaCl salt near 1310 cm^−1^, 1110 cm^−1^, and 1640 cm^−1^ in the SCM spectra, which is similar to the results of Varnaitė-Žuravliova’s study [25]. The spectra of SCM showed the same peaks as those of CM, indicating that the addition of salt to the electrospinning solution of chitosan did not change the chemical composition of the electrospun chitosan film.

### 2.4. X-Ray Diffraction (XRD)

Figure 3c displays the XRD spectra of both CM and SCM (with 1% (*w*/*v*) NaCl). In both spectra, a broad peak was observed at 2θ = 20°, which is characteristic of partially crystalline forms of chitosan, including both anhydrous and hydrated crystalline structures [14]. When comparing with the XRD spectrum of sodium chloride crystals, no distinct characteristic peaks of NaCl are present in the SCM spectrum, indicating the absence of salt crystals in the SCM [44]. This result further supports the FTIR analysis, confirming that the addition of salt to the spinning solution did not alter the chemical composition of the chitosan nanofibers.

### 2.5. Water Retention

The water retention behavior of the membranes reflects their ability to absorb exudates from wounds. The abundant micropores within the membranes enhance water absorption by increasing surface contact with water, while also providing favorable conditions for cell adhesion and spreading [45]. As shown in the results, the water retention rate of SCM (736.81 ± 37.89%) was significantly higher than that of CM (372.97 ± 11.22%) after 0.5 h and remained stable thereafter (Table 1). Notably, both membranes exhibited minimal expansion in length, 102.79 ± 1.17% for CM and 97.24 ± 0.83% for SCM, indicating that the shape of both membranes remained largely unchanged after water absorption. SCM exhibited initial swelling and thickening during the early stages of water absorption, leading to a slight reduction in side length. FTIR and XRD analysis confirmed no significant differences in the chemical structures of the two membranes. Therefore, the observed variation in water retention may be attributed to differences in fiber diameter. As the fiber diameter decreases, the surface area-to-volume ratio increases, leading to a larger nanoporous space. This allows SCM to have more extensive contact with water, resulting in greater water absorption and swelling [46], see more in Supplementary Materials.

### 2.6. Tear Strength

The suture pull-out test was used to evaluate the tear strength, indicating the handleability of the membrane in clinical operations. The results showed that the tear strength of CM and SCM (with 1% (*w*/*v*) NaCl) was 1.63 N/mm and 1.72 N/mm, respectively (Figure 3d). There was no significant difference (*p* > 0.05), indicating that adding NaCl solution before chitosan electrospinning did not affect the mechanical properties of the chitosan membrane, see more in Supplementary Materials.

### 2.7. Degradation

The degradation of the membranes was evaluated to assess their breakdown rate when implanted in the human body. In tissue engineering applications, the degradability of electrospun chitosan membranes is advantageous, as it eliminates the need for a second surgery and reduces unnecessary tissue damage. The results showed that both CM and SCM degraded significantly after 5 days, with substantial weight loss (Figure 3e). The degradation rate of CM was consistent with the previous study [47], while SCM exhibited a significantly faster degradation rate. This accelerated degradation could be attributed to the thinner fibers in SCM, which may be more susceptible to breakdown.

### 2.8. Cell Culture

**Figure 4:**
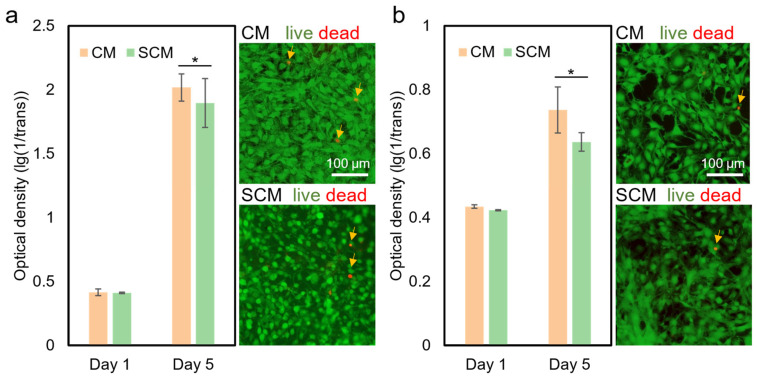
Cell culture results of CM and SCM (with 1% (*w*/*v*) NaCl). (**a**) Proliferation of fibroblasts (NIH3T3 cells) on CM and SCM (left), with live/dead staining images on the 5th day (right). (**b**) Proliferation of osteoblasts (MC3T3E1 cells) on CM and SCM (left), with live/dead staining images on the 5th day (right). * denotes a significant difference (*p* < 0.05). “CM” represents “chitosan membrane” and “SCM” represents “chitosan membrane with salt”. Arrows indicate dead cells.

The cell culture experiment was conducted to evaluate the biocompatibility of CM and SCM (with 1% (*w*/*v*) NaCl) after TEA/tBOC treatment. After 5 days of culture, both CM and SCM were proved to be biocompatible with fibroblasts (NIH3T3, Figure 4a) and osteoblasts (MC3T3E1, Figure 4b). Both cell types showed significant proliferation (*p* < 0.05) on CM and SCM from day 1 to day 5, similar to previous studies [14,17]. There was no significant difference (*p* > 0.05) between the two types of membranes at each time point in both the fibroblast and osteoblast cell culture experiments. Live and dead staining graphs showed no obvious dead cells on the 5th day for both CM and SCM, indicating a high cell survival rate. These results demonstrate that adding 1% (*w*/*v*) NaCl did not affect the biocompatibility of electrospun chitosan membranes, see more in Supplementary Materials. 

In previous studies, NaCl was also a commonly used additive in polymer electrospinning solutions and was proven to be biocompatible with cells [48,49]. MG63 cells proliferated significantly after 11 days on a chitosan/PEO scaffold that was electrospun with 0.2% NaCl. Additionally, smooth muscle cells showed significantly proliferation after 14 days on collagen/elastin membranes that were electrospun with 0.5% (*w*/*v*) PEO and 42.5 mM NaCl [45]. These studies demonstrate that the addition of a small amount of sodium chloride does not affect the biocompatibility of electrospun membranes.

## 3. Materials and Methods

### 3.1. Chitosan Solution and Membrane Preparation

The electrospun chitosan solution was prepared by dissolving 550 mg of chitosan (70% DDA, MW = 1,000,000, Meryer (Shanghai) chemical technology company, Shanghai, China) in 7 mL of TFA (Shanghai Macklin biochemical technology company, Shanghai, China) and 3 mL of dichloromethane (DCM, Shanghai Macklin biochemical technology company, China) overnight. The salt solution was prepared by dissolving 100 mg of sodium chloride (NaCl, 99% purity, Shanghai Aladdin biochemical technology company, Shanghai, China) in 300 μL of deionized (DI) water. The chitosan solution with NaCl was prepared just before the electrospinning by adding the required amount of salt solution to the prepared electrospun chitosan solution. 

Electrospinning of the CM was based on a previously reported method [14,17]. After filling the electrospun solution into a 10 mL syringe, a high voltage of 26 kV was applied to the needle tip. An aluminum foil-covered round plate, rotating at a speed of 8.4 rpm, was placed 15 cm from the needle tip.

After collecting electrospun membranes, a post-electrospinning treatment involving TEA and tBOC, innovated in a previous study, was used to neutralize the membranes [15]. Membranes were immersed in a 10% (*w*/*v*) solution of TEA (Shanghai Macklin biochemical technology company, China)/tetrahydrofuran (THF, Shanghai Macklin biochemical technology company, China) for 24 h under mild magnetic stirring to remove the TFA salt. After rinsing the membranes with pure THF twice, the membranes were immersed in a 0.1 g/mL solution of tBOC (Shanghai Aladdin biochemical technology company, China)/THF for 24 h. After washing the membranes with pure THF three times, they were dried between two pieces of nylon mesh in the air to keep them flat.

### 3.2. Solution Conductivity

The conductivity of 30 mL chitosan solutions (n = 3) was evaluated by electrochemical impedance spectroscopy (EIS) with the electrochemical workstation (CHI 660D, Chenhua, China). The testing electrodes (Wuhu keying new material corporation, Wuhu, China) were selected following the three-electrode system, which included a glassy carbon electrode for the working electrode, a platinum electrode for the counter electrode, and a mercurous sulphate electrode for the reference electrode. The frequency range of EIS measurements was set to 10 to 10^5^ Hz, with the voltage of 5 mV.

### 3.3. Solution Viscosity

The viscosity of chitosan solutions (n = 3) was tested by a glass capillary viscometer (Shanghai Baoshan Qihang glass instrument factory, China) at room temperature. After recording the flowing time between the two markers (*t*), the dynamic viscosity of the tested solution was calculated using Equation (1):

(1) η=C×t×ρ

where *η* (mPa·s) represents the dynamic viscosity, *C* (mm^2^/S^2^) denotes the viscosity number, and *ρ* (g/cm^3^) indicates the solution density.

### 3.4. Scanning Electron Microscopy (SEM)

The nanostructure of the two types of membranes was examined using an SEM (MIRA LMS, TESCAN, Tempe, AZ, USA). The membranes were attached to an SEM stub and coated with an 8 nm layer of gold-palladium. They were then examined at 2500× magnification. In each sample, more than 20 fiber diameters were measured.

### 3.5. Fourier Transform Infrared Spectroscopy (FTIR)

FTIR spectra were collected to evaluate the chemical structure of SCM and CM using a Nicolet^TM^ iS20 FTIR spectrometer (Thermo Fisher Scientific, Lenexa, KS, USA). Spectra were collected by scanning samples from 500 cm^−1^ to 4000 cm^−1^ for 32 scans each.

### 3.6. X-Ray Diffraction (XRD)

XRD analysis was conducted to assess the crystallinity of the chitosan membranes. Both CM and SCM were first immersed in liquid nitrogen and then ground into fine powders using a mortar and pestle. The samples were subsequently analyzed using an automated multipurpose X-ray diffractometer (Ultima IV, Rigaku, Tokyo, Japan) operating in grazing incidence mode with a wavelength of 1.54 Å. Data collection was performed over a 2θ range of 10° to 80°.

### 3.7. Water Retention

Water absorption was evaluated to determine the swelling extent and water retention capacity of the membranes. Both CM and SCM were cut into 3 cm × 2 cm rectangle. The samples were then immersed in DI water, and the edge length and weight were measured at specific time intervals. The initial edge length and weight were recorded as “*l*_0_” and “*w*_0_”, respectively. At each time point, the samples were taken out and the excess water was removed using filter paper. The edge length of samples was recorded as “*l_t_*” and the weight as “*w_t_*”. The membrane swelling length was calculated using Equation (2), and the water retention content was calculated using Equation (3):

(2) $$membrane\;swelling\;length\;\left(\%\right)=\;\frac{l_{t}}{l_{0}},$$

(3) $$water\;retention\;content\;\left(\%\right)=\frac{m_{t}}{m_{0}}$$

### 3.8. Tear Strength

The tear strength of CM and SCM (n = 3/membrane type) treated with TEA/tBOC was evaluated using an electronic universal testing machine (STS20K, Xiamen Yishite Instruments, Xiamen, China) as an indicator of clinical operability.

Each sample was cut into a 10 × 40 mm^2^ rectangle, and surgical sutures were passed through one end at a point 5 mm from the wide edge of the sample and 5 mm from the long edge. The upper clamp of the tension testing machine was used to secure the surgical sutures, while the lower clamp was used to secure the other end of the sample. The load cell used was 50 N, and the extension rate was 10 mm/min. The maximum load was recorded in Newtons (N) and then normalized to film thickness. Three samples of each membrane were tested.

### 3.9. Degradation

The degradation of the membranes was assessed by measuring mass loss over time. Membranes were cut into 3 cm × 2 cm rectangle and immersed in phosphate-buffered saline (PBS) solution (Beijing Solarbio Science & Technology Co., Beijing, China) containing 100 μg/mL lysozyme (Beijing Solarbio Science & Technology Co., China), and a 5% penicillin–streptomycin–gentamicin solution (Beijing Solarbio Science & Technology Co., China). At each time point, the membranes were removed from the solution, rinsed with DI water, dried overnight at 60 °C, and weighed to determine the change in mass. After weighing, the membranes were returned to the solution to continue the incubation period. A higher concentration of lysozyme was used in this experiment compared to the physiological levels found in human plasma (3–8 μg/mL) to accelerate the degradation process.

### 3.10. Cell Culture

After high-pressure steam sterilization, round CM and SCM samples (*n* = 3/membrane type, diameter = 1 cm) were placed into the 48-well plate. Fibroblasts (NIH3T3 cells) and osteoblasts (MC3T3E1 cells) were seeded on the membrane, respectively. The CM and SCM samples were rinsed in culture media and then seeded with cells at 2 × 10^4^ cells/well. Cells were grown in DMEM media supplemented with 10% FBS and 1% penicillin/streptomycin. At the required time point, the media was removed, and each well was cleaned with PBS three times. Subsequently, 500 μL of media containing 10% CCK-8 were added to each well, and the plate was cultured in a constant temperature incubator at 37 °C with 5% CO_2_ for 2 h. Afterward, 100 μL of the media was transferred into a new 96-well plate, and the absorbance at 450 nm was measured with a microplate reader (SPARK 10M, TECAN, Tempe, AZ, USA). The absorbance value is proportional to the number of cells. The survival rate and morphology of the cells on the fifth day were observed by inverted fluorescence microscopy and staining with Calcein acetoxymethyl ester (Calcein-AM)/propidium iodide (PI).

### 3.11. Statistical Analysis

A single factor analysis of variance (ANOVA) was used to analyze the results of solution viscosity and fiber diameter. An independent sample *t*-test was used to analyze the results of mechanical strength, while a two-way ANOVA was employed for analyzing solution conductivity, water retention, degradation, and cell proliferation.

## 4. Conclusions

In this study, the addition of NaCl to chitosan solutions during electrospinning significantly improved spinnability and reduced the limitations posed by environmental humidity. The incorporation of NaCl led to an increase in solution conductivity and a decrease in viscosity, facilitating the electrospinning process. Comparative analysis of the physicochemical properties of CM and SCM revealed that the addition of 1% (*w*/*v*) NaCl did not alter the chemical groups, mechanical strength, or in vitro cell proliferation characteristics of the membranes. However, the reduced fiber diameter in SCM may contribute to its higher water retention capacity and faster degradation rate. These results suggest that 1% (*w*/*v*) NaCl serves as an effective additive to enhance the electrospinning of chitosan in low-humidity environments, optimizing fiber formation while maintaining key physicochemical properties. This study addresses the challenge of humidity limitations in electrospinning chitosan using the TFA/DCM solvent system, bridging the gap in current advancements within the field.

## Figures and Tables

**Table 1:** CM and SCM membrane swelling extent and water retention content.

Hours (h)	Membrane Swelling Length (%)		Water Retention Content (%)	
	CM (mean ± std)	SCM (mean ± std)	CM (mean ± std)	SCM (mean ± std)
0	100 ± 0	100 ± 0	100 ± 0	100 ± 0
0.5	102.79 ± 1.17 *	97.24 ± 0.83 ^+^	372.97 ± 11.22 *	736.81 ± 37.89 ^+^
2	102.2 ± 1.35 *	97.61 ± 0.95 ^+^	383.91 ± 2 *	743.39 ± 39.6 ^+^

* and ^+^ denote significant differences (*p* < 0.05).

## Data Availability

The data presented in this study are available on request from the corresponding author.

## Supplementary Materials

The following supporting information can be downloaded at: https://www.mdpi.com/article/10.3390/md22100443/s1.
