# Supplementary figures and images for: Enhancing Electrospinnability of Chitosan Membranes in Low-Humidity Environments by Sodium Chloride Addition
Source: Mar Drugs. 2024 Sep 27;22(10):443. doi: 10.3390/md22100443 (PMC11509170; doi:10.3390/md22100443)

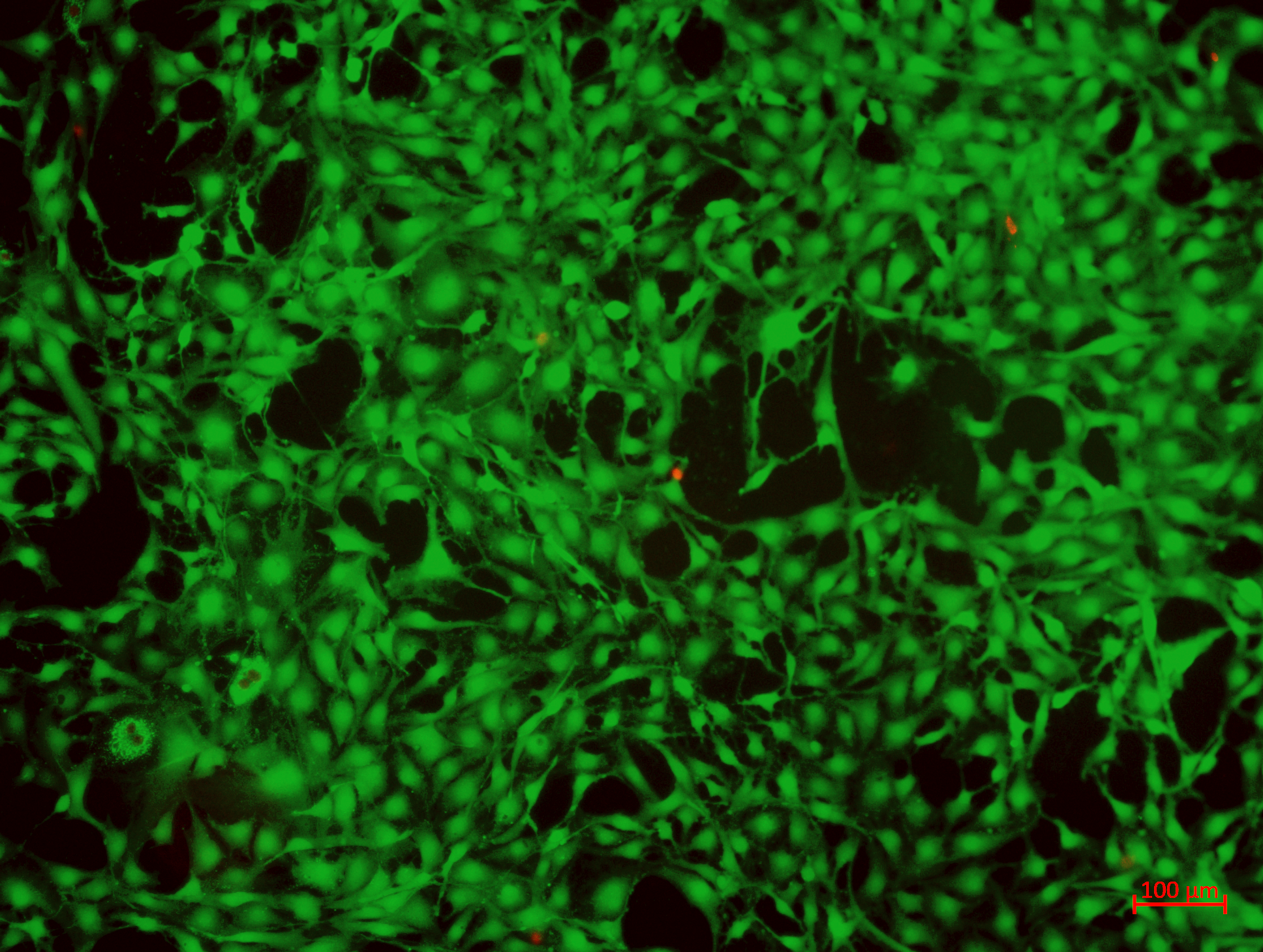

Supplement: Supplementary file 1 [file marinedrugs-22-00443-s001.zip › Cell culture/ld-MC3T3E1-TEAtBOC-treated CM.tif]

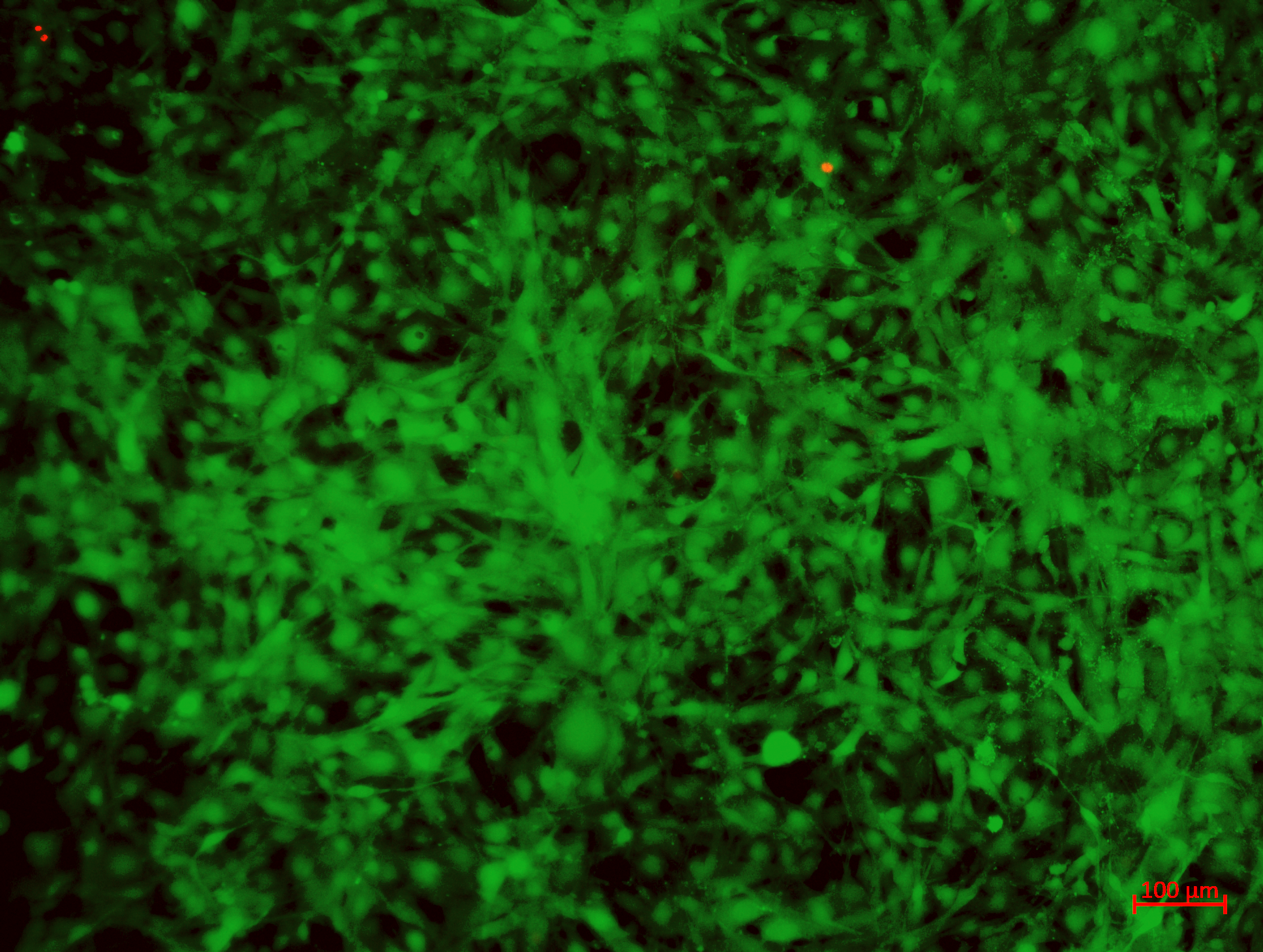

Supplement: Supplementary file 1 [file marinedrugs-22-00443-s001.zip › Cell culture/ld-MC3T3E1-TEAtBOC-treated SCM.tif]

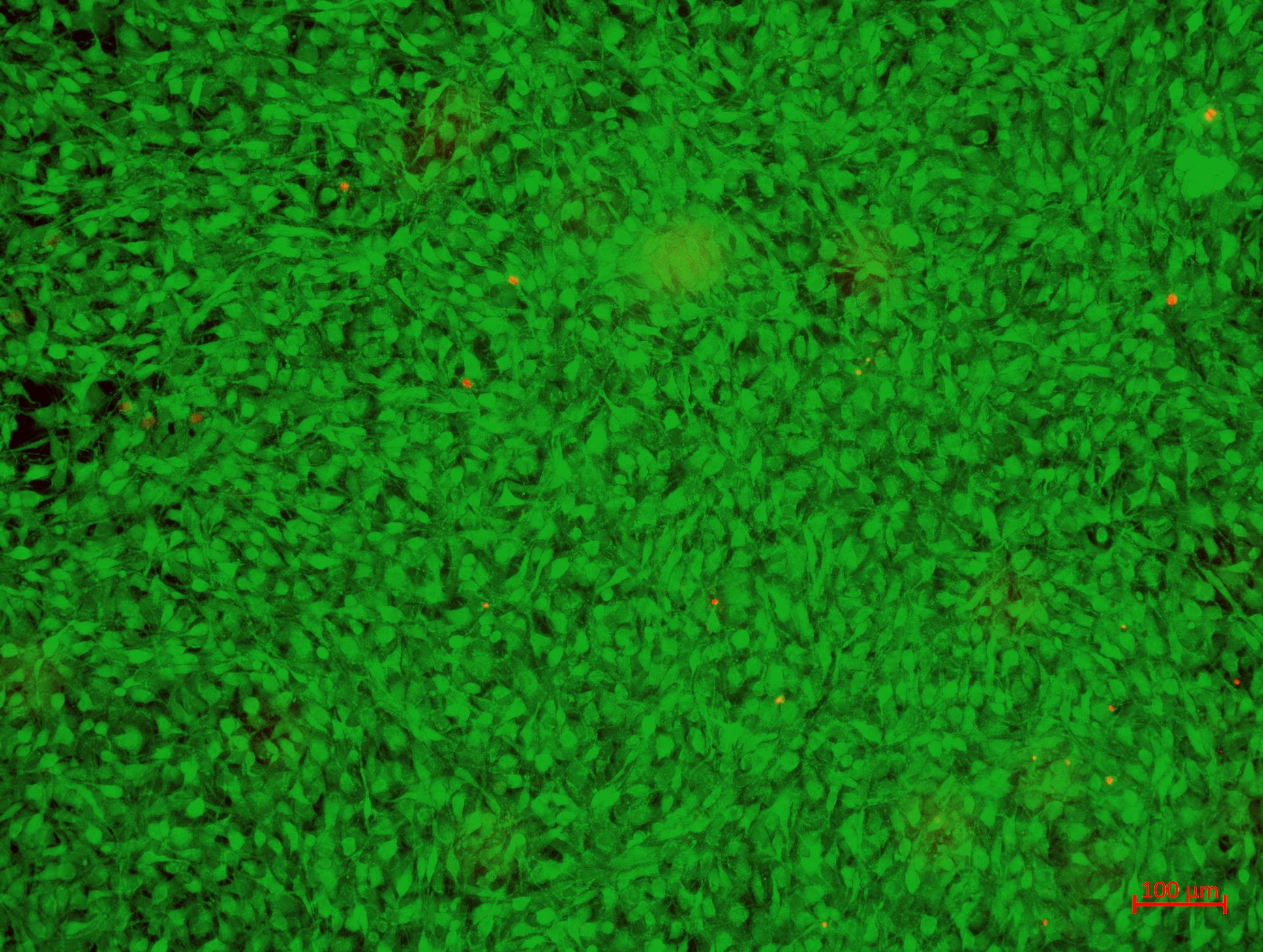

Supplement: Supplementary file 1 [file marinedrugs-22-00443-s001.zip › Cell culture/ld-NIH3T3-TEAtBOC-treated CM.tif]

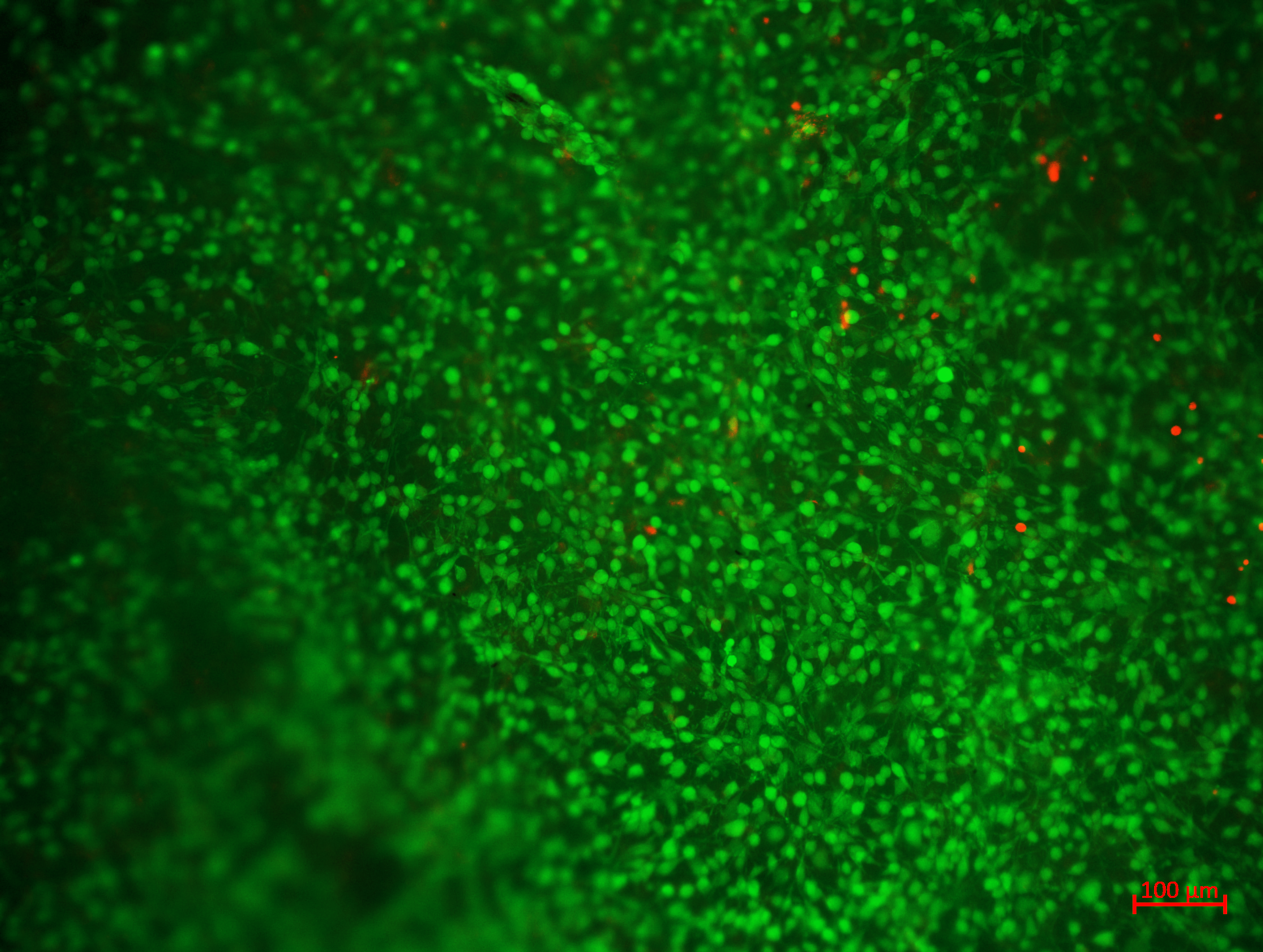

Supplement: Supplementary file 1 [file marinedrugs-22-00443-s001.zip › Cell culture/ld-NIH3T3-TEAtBOC-treated SCM.tif]

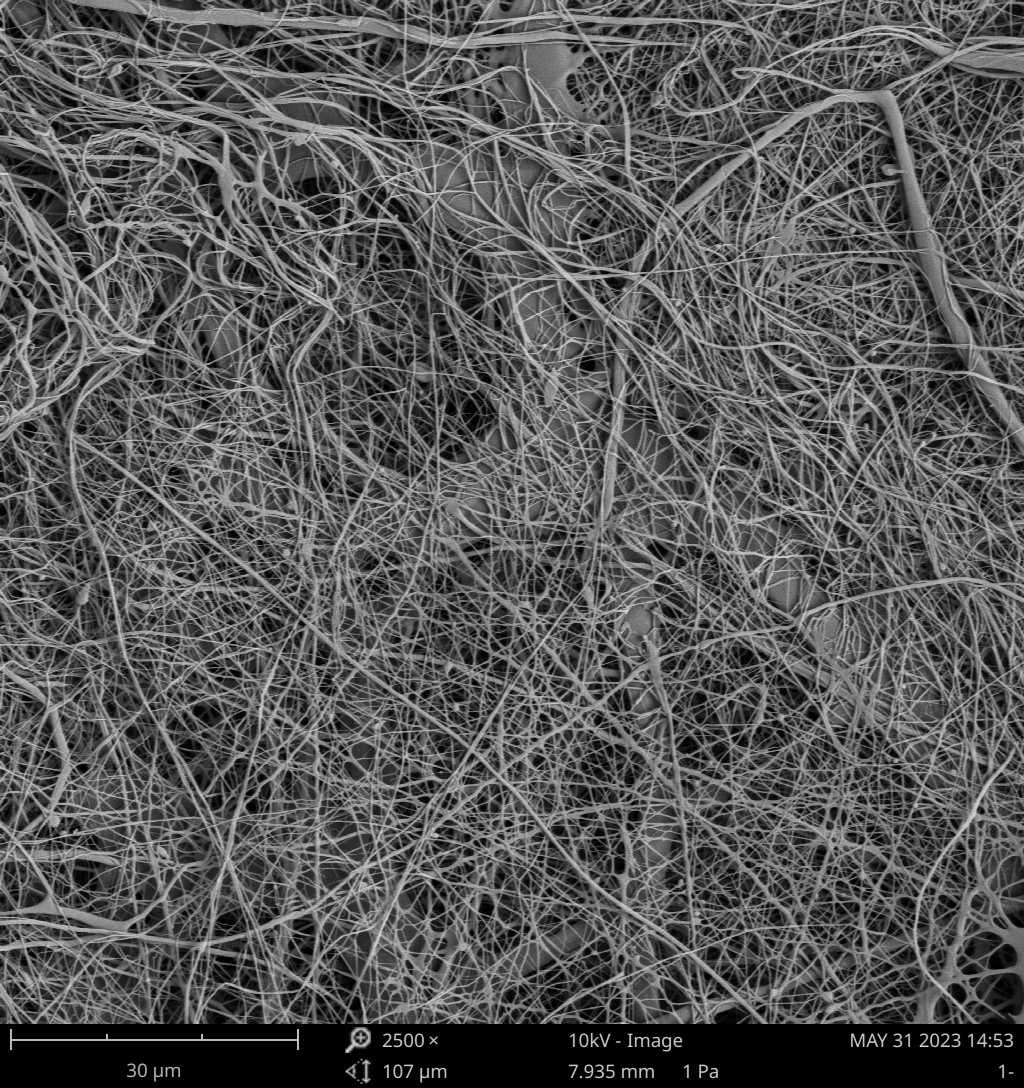

Supplement: Supplementary file 1 [file marinedrugs-22-00443-s001.zip › SEM/non-treated CM.tiff]

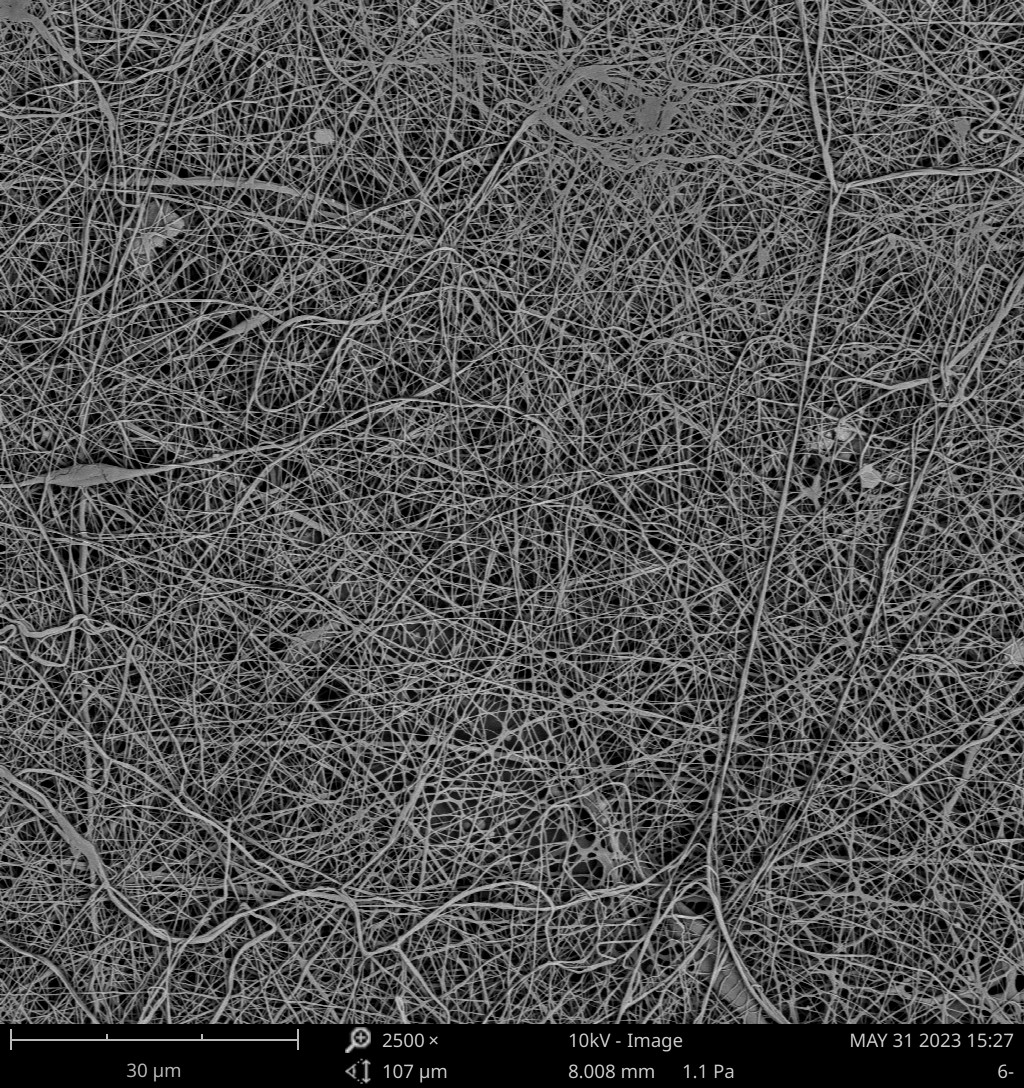

Supplement: Supplementary file 1 [file marinedrugs-22-00443-s001.zip › SEM/non-treated SCM w 100mg NaCl.tiff]

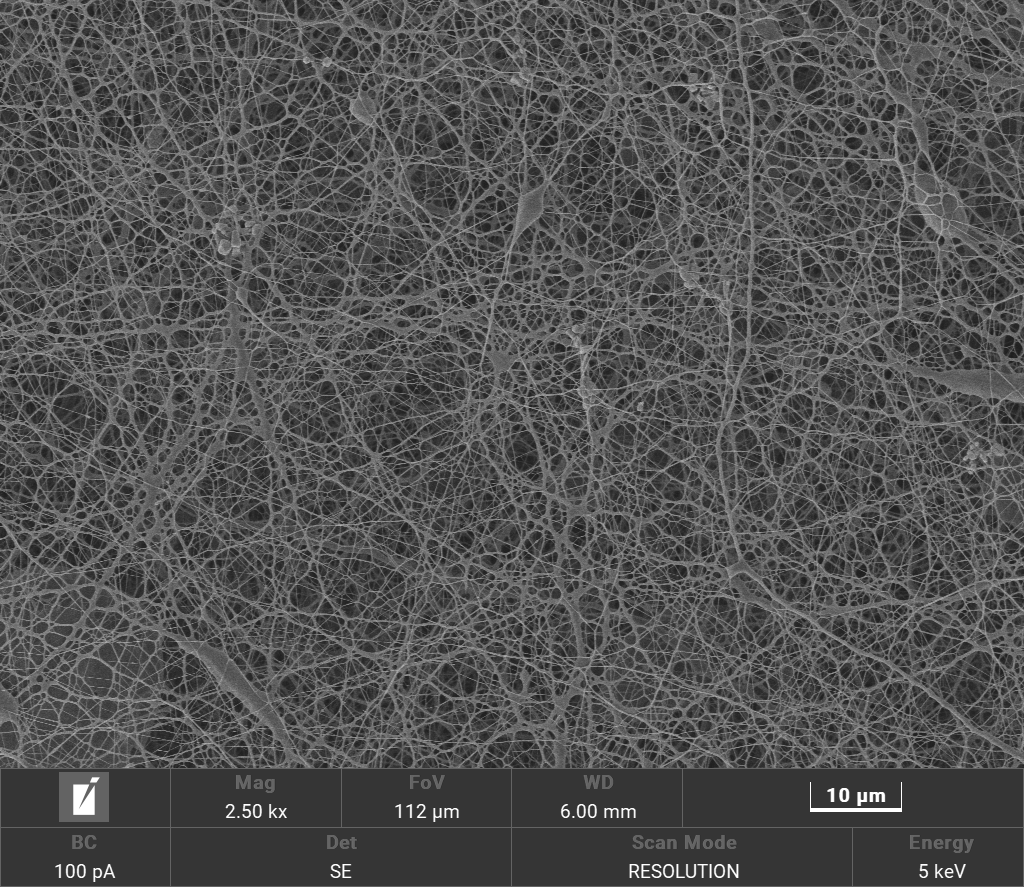

Supplement: Supplementary file 1 [file marinedrugs-22-00443-s001.zip › SEM/non-treated SCM w 200mg NaCl.tif]

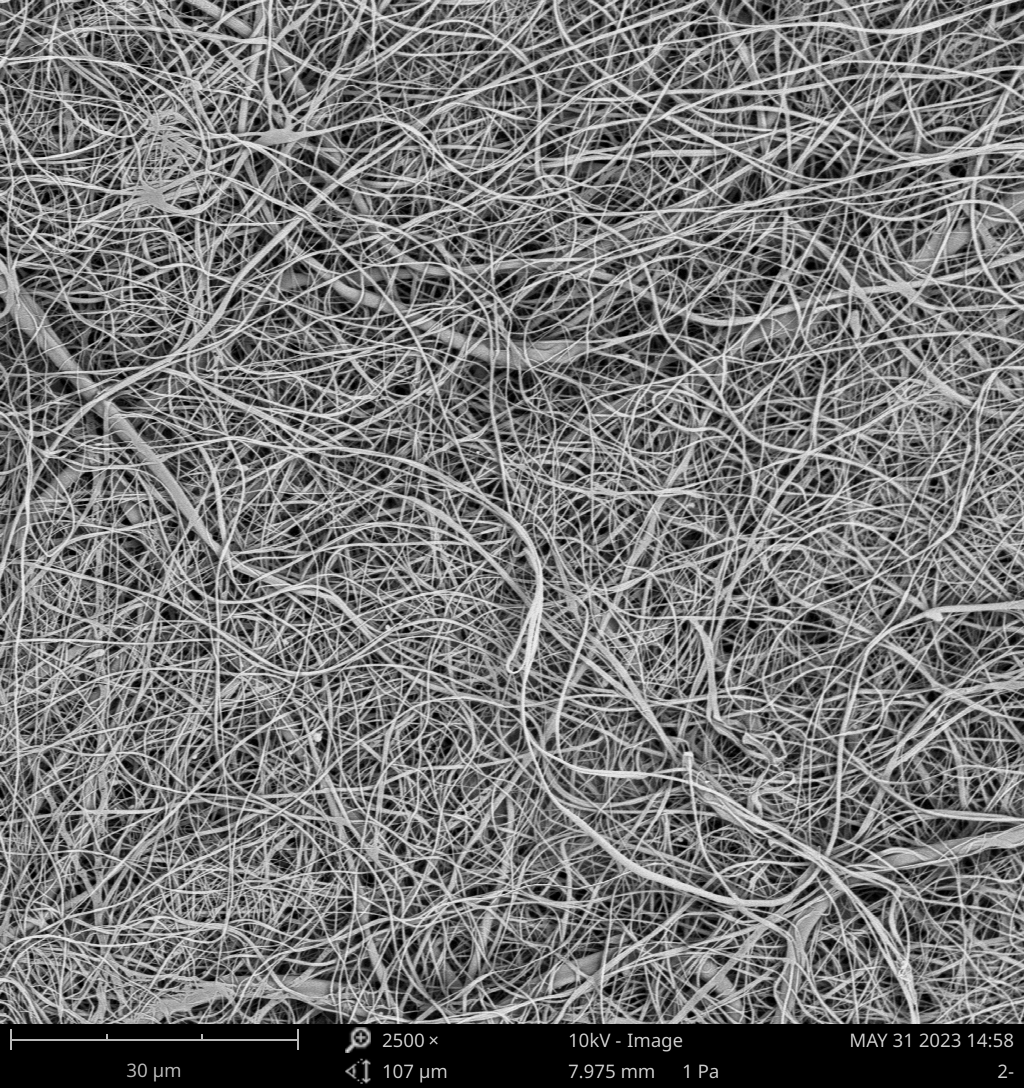

Supplement: Supplementary file 1 [file marinedrugs-22-00443-s001.zip › SEM/TEAtBOC-treated CM.tiff]

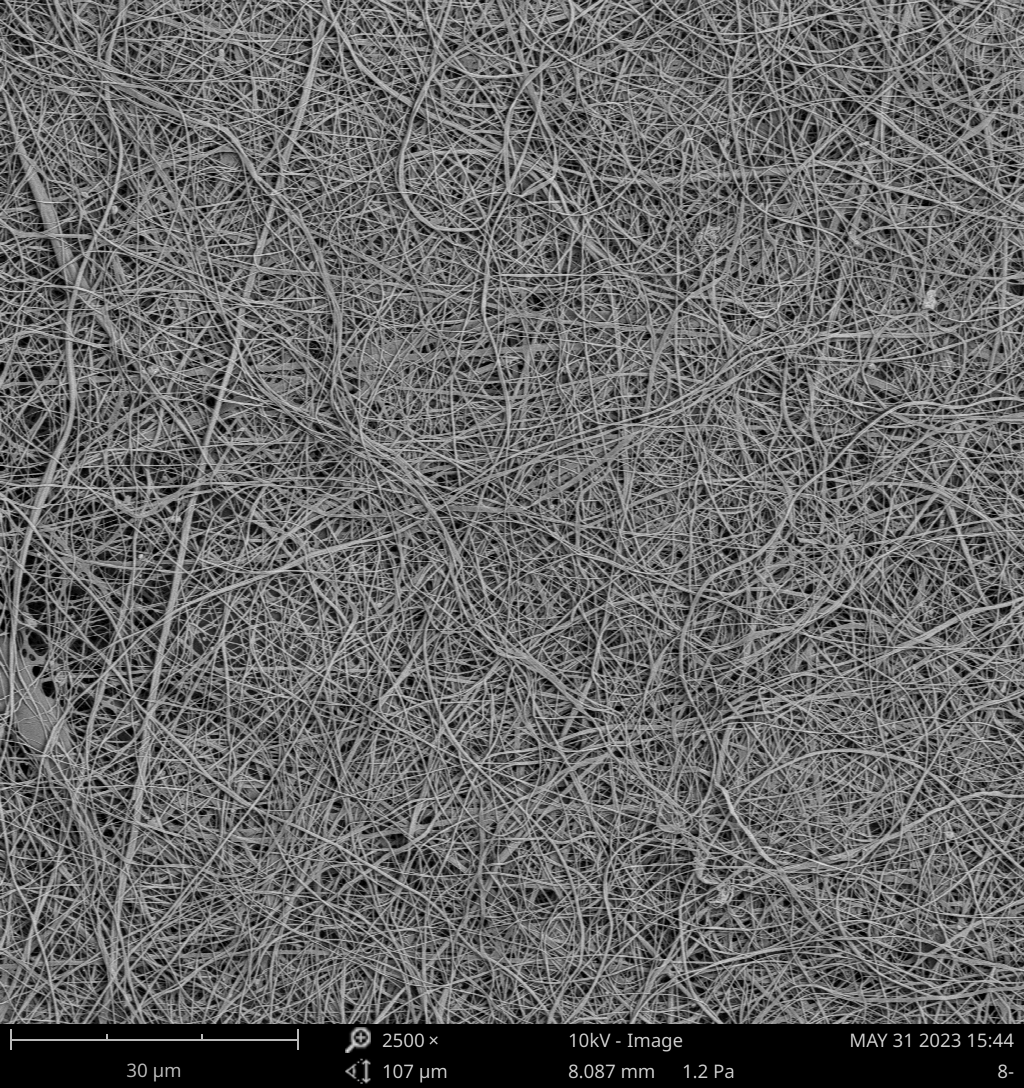

Supplement: Supplementary file 1 [file marinedrugs-22-00443-s001.zip › SEM/TEAtBOC-treated SCM w 100mg NaCl.tiff]

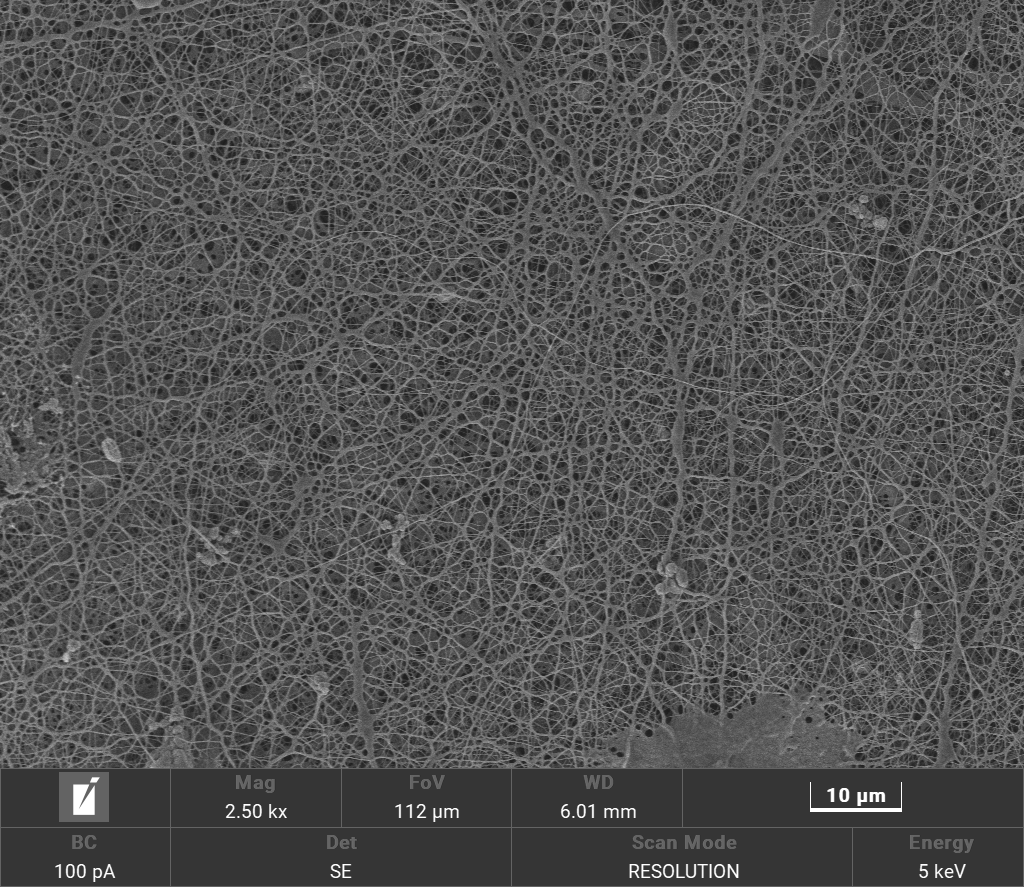

Supplement: Supplementary file 1 [file marinedrugs-22-00443-s001.zip › SEM/TEAtBOC-treated SCM w 200mg NaCl.tif]
